# Seeking help for depression from family and friends: A qualitative analysis of perceived advantages and disadvantages

**DOI:** 10.1186/1471-244X-11-196

**Published:** 2011-12-15

**Authors:** Kathleen M Griffiths, Dimity A Crisp, Lisa Barney, Russell Reid

**Affiliations:** 1 Centre for Mental Health Research, The Australian National University, Acton, Canberra, ACT, 0200, Australia; 2 Directorate of Mental Health, Department of Defence, CP2-7-043A, Canberra, ACT, 0200, Australia

## Abstract

**Background:**

People with depression often seek help from family and friends and public health campaigns frequently encourage such help seeking behaviours. However, there has been little systematically collected empirical data concerning the effects of such informal help seeking. The current study sought to investigate the views of consumers about the advantages and disadvantages of seeking support from family and friends for depression.

**Methods:**

Participants were the subset of 417 respondents to a survey, sent to 7000 randomly selected members of an Australian electoral community, who indicated that they had sought help for depression from family or friends. One item on the survey asked participants to indicate the advantages or disadvantages of seeking help from family or friends. A coding system was developed based on a content analysis of the responses to the item. Each of the responses was then coded by two raters.

**Results:**

Respondents identified both advantages and disadvantages of seeking support from friends. The most commonly cited advantage was social support (n = 282) including emotional support (n = 154), informational support (n = 93), companionship support (n = 36) and instrumental support (n = 23). Other advantages related to family's or friend's background knowledge of the person and their circumstances (n = 72), the opportunity to offload the burden associated with depression (n = 62), the personal attributes of family and friends (n = 49), their accessibility (n = 36), and the opportunity to educate family and friends and increase their awareness about the respondent's depression (n = 30). The most commonly cited disadvantages were stigma (n = 53), inappropriate support (n = 45), the family member's lack of knowledge, training and expertise (n = 32) and the adverse impact of the help seeking on the family/friend (n = 20) and the relationship (n = 18).

**Conclusions:**

Family and friends are well placed to provide support which consumers perceive to be positive and which can assist them in obtaining formal mental health treatment. However, the input of some family members may be unhelpful or toxic. There may be benefits in undertaking community education and destigmatisation programs which target carers.

## Background

Members of the public frequently perceive sources of informal help (e.g., family and friends) as useful for dealing with depression [1-5]. Indeed, some studies have reported that the public prefers such help ahead of support from formal sources [1,2] and particularly formal mental health sources [6]. Moreover, there is some evidence that perceived positive support from friends and family members or other informal sources is associated with improved recovery among consumers with a depressive disorder [7,8]. It is perhaps not surprising then that help seeking from family and friends is often encouraged in mental health promotion campaigns.

However, some researchers have expressed the concern that informal help seeking may be unhelpful or harmful [5,9,10]. For example, Rickwood [9] hypothesised that the mental health problems of adolescents may be amplified if it results in mutual disclosure, unhelpful interpretations and rumination. Christensen et al. [10] found that an internet psycho-educational intervention which was effective in reducing depressive symptoms did not increase evidence-based help seeking but was associated with a reduction relative to a control group in seeking help from family and friends. They suggested that these informal sources of help may be "unhelpful, even toxic under certain circumstances" and that "the website may have served to reduce inappropriate help seeking actions" (p.7).

However, there has been little systematically collected empirical data concerning the effects of seeking help for depression from family or friends. Vollmann and his collaborators have recently studied the anticipated helpfulness for depression of several different types of social support from a friend [11]. People with depression considered that emotional support would be most helpful, followed by instrumental (tangible) support and informational support. Further, participants with depression anticipated that protection oriented support (shielding the person with depression from unwanted input) would be more helpful than activation support (facilitating activities). However, the Vollmann et al. study did not evaluate *actual *(as opposed to anticipated) experiences of support. Nor did they investigate the *disadvantages *of seeking support from friends or family which as noted above may yield negative mental health outcomes. Furthermore, the study only investigated a restricted and pre-specified range of support behaviours. These behaviours were not formulated from the self-reported experiences of consumers. In addition, participants were recruited from a psychiatry centre and a depression self help group and therefore the views of these consumers may not be representative of those in the general community, a substantial percentage of whom do not seek professional help [12]. Finally, the Vollmann et al. study was restricted to consumer perceptions of help seeking from friends.

To the best of our knowledge, there have been no studies of consumer experiences of informal help seeking based on categories derived from consumer reports. Nor has any study investigated the disadvantages of such help seeking, consumer experiences of actual as opposed to anticipated help seeking or consumer experiences of help seeking from family members. Accordingly, the current study sought to elicit and undertake a qualitative analysis of the views of a large community-based sample of consumers about the advantages and disadvantages of seeking help from informal sources, specifically support sought from family and friends.

## Methods

### Measures

This study was part of a larger project in which participants completed a 'Beliefs about Depression and Help Seeking Questionnaire'. This self-report survey comprised measures of demographic status, personal and perceived stigma, help-seeking intention and behaviours, self reported depressive symptoms (PRIME MD) and previous personal experience of depression (see [13]). In addition, respondents with a past history of depression were asked to respond to the open-ended question: *"If you sought support from friends and or/family, what would you say were the advantages and/or disadvantages of doing this?" *The current paper is concerned with responses to this open ended item. Ethics approval was obtained for the study from the Australian National University Committee for Ethics in Human Research.

### Participants

Seven thousand residents, randomly selected from the electoral roll for a New South Wales electorate in Australia were sent the questionnaire by post. Registration on an electoral roll is compulsory in Australia. Of the 1312 respondents who completed the questionnaire, 722 (55%) reported having personally experienced depression. Of these, 57.8% (n = 417) had both sought help from informal sources and provided a response to the open-ended question which is the focus of this paper. The age of the latter respondents ranged from 18 to 84 years (mean = 48.6, SD = 14.2), 22.1% (n = 92) held a Bachelor's degree or higher educational qualification and 69.3% (n = 289) were women, the latter being consistent with the gender distribution of depression in the general population [14].

### Analyses

The 417 open-ended responses were subjected to a qualitative content analysis. Initially, two raters (KG and DC) independently read and categorised response into categories and subcategories under the general headings of 'Advantages' and 'Disadvantages' of seeking support from friends or family. The two raters then met, and following discussion agreed on a final coding system. Each participant response was then coded independently using NVivo8 qualitative data analysis software [15] by two raters (DC, RR), one of whom (RR) had not been involved in the development of the coding system. Discrepancies were subsequently resolved by discussion between the two raters. The responses of each individual could be allocated to more than one category or subcategory. In addition, following the content analysis, two raters (KG and RR) independently coded each response as describing either 'advantages only', 'disadvantages only' or both 'advantages and disadvantages' of consulting a family or friend. Again, any discrepancies were resolved by discussion between the raters.

## Results

Of the 417 participants in this study, 240 (51.3%) cited only advantages of consulting a family or friend, 25 (6%) reported disadvantages only and 138 (33.1%) described both disadvantages and advantages. Thus 84.4% of respondents reported at least one advantage of informal help seeking and 39.1% reported at least one disadvantage. Fourteen responses (3.4%) could not be coded unambiguously into any of these categories. Ten of the participants qualified their responses, indicating that the answer depended on the context or the person involved. The reported advantages, followed by the reported disadvantages of consulting family and friends about depression are described below.

### Advantages

As shown in Table 1, six main categories emerged with respect to the reported advantages of seeking help from family and friends for depression. These were, in order of frequency of comments: (i) the social support they provided (n = 282); (ii) their background knowledge (n = 72); (iii) the opportunity to offload the burden associated with depression (n = 62); (iv) their personal attributes (n = 49); (v) their accessibility (n = 36); and (vi) the opportunity to educate family and friends and increase their awareness of the respondent's depression (n = 30). Each main category comprised a number of subcategories. Some subcategories were further coded into different types of advantage; these are referred to in italics below.

**Table 1:** Frequency (n) of respondents citing advantages of seeking support for depression from family and friends

Categories and subcategories	n	Coded Types
**1. Support**	**282**	
Emotional	154	Understanding (n = 55), love (22)*, care (20), privacy and confidentiality (18), trust (10), sympathy (8), acceptance (8), encouragement (9), reassurance (7), empathy (6), self-esteem support (6), strengthened relationship (6), comfort (4), feeling of safety (4), compassion (3), hope (3), patience (3), consideration (2), empowerment (2), familiarity (2), intimacy (2), kindness (2), prayer (2), concern (1)
Informational	93	Advice (37), facilitation of help seeking (31), appraisal support (26), sharing of experiences (14)
Companionship	36	Sense of connection (14), humour (4)
Instrumental	23	Practical support (13), expressed willingness to take action (9)
Universality	7	
**2. Knowledge of consumer**	**72**	Personal attributes (50), circumstances (40)
**3. Enabled burden offloading**	**62**	
**4. Positive personal attributes**	**49**	Trustworthiness (17), non-judgmental attitude (15), genuine interest in well-being (7), genuinely caring (5), honesty (4), good listener (4), loving (3)
**5. Accessible and acceptable**	**36**	Available when needed (9), available to discuss matters honestly (7), comfortable talking to family/friends (5), can be yourself (4), more personal (3), approachable (3), less embarrassing (3), less shame (2), no cost (2), no time limit (1)
**6. Educate family/friend**	**30**	
**7. Other**	**31**	Listening ear (18), general-e.g., you feel better (13), had professional knowledge-experience (9), openness/self-disclosure (5)

*Numerals in brackets refer to number of participants citing type of advantage.

### (i) Category 1-Social support

The most commonly cited advantage of talking to family and friends was 'support'. Some respondents mentioned support only in a general sense without specifying its precise nature (e.g., "My husband was very supportive"). However, most respondents described this support more specifically. The most frequently cited form of support was emotional, followed by informational, companionship, and instrumental support (see Table 1).

#### Emotional support

Several types of emotional support were valued by consumers. The most commonly cited of these was the emotional *understanding *that family and friends provided. Related to this, but less commonly cited were the *empathic support*, *sympathy*, and *compassion *that informal sources provided. Such help was sometimes seen as distinctly characteristic of informal sources as illustrated by a respondent who commented: "Generally family is most caring and compassionate". Similarly, *acceptance *was cited by some respondents as a helpful attribute of family and friends. One participant noted: "Friends were a huge support to me which is what I needed in order to talk out what was going on inside of me as I worked to help myself; it was their acceptance of me as a person that was most appreciated and affirming".

*Love *and a demonstration of *caring *were other commonly reported forms of emotional support provided by family and friends. Respondents cited feelings of 'renewed love', 'unconditional love', and a realisation of how much people loved and cared about them. One respondent commented: "My family and close friends really helped me a lot. I found it much easier to cope when I knew that I had a support network around me that cared for me". Similarly, *kindness *of family or close friends and *intimacy *or *closeness *associated with the relationship were reported to be an advantage of seeking informal support.

*Privacy and confidentiality *as well as *trust *were cited as important advantages of seeking support from family or friends. In some cases these factors determined which friend or family members a participant was willing to approach for support. For example, one participant remarked: "The only person I sought help from was my husband as I knew he would not tell everyone he met". Another commented: "The need to feel complete trust is essential".

The fact that support was provided by a *familiar *person was also seen as an advantage. For example, one respondent noted: "It was confidential and familiar and not cold and clinical as I would imagine the professionals are". Involving family also invoked a sense of *safety *or protection in some respondents. For example, various respondents reported that it was 'secure and safe' to be 'exposed' to family and friends, that they would protect them "from stressful social situations" and that they can "watch out for you". In addition, some participants appreciated being treated *patiently *and a number reported that their family offered them *encouragement *and *reassurance *throughout their experience. As one respondent noted: "I found that they understood what I was going through and encouraged me to ... do something to help myself by taking time out for my own interests". Another noted: "The advantage was the reassurance I received and knowing I could get help and that depression was brought on by circumstances beyond my control/and that someone cared and understood my position". The *comfort *provided by family and friends was another reported advantage of seeking informal support.

Informal support was also reported to improve *self-esteem*. For example, one respondent stated that talking to family and friends "made me feel good about myself" and another that it helped to "overcome feelings of loneliness, inadequacy, of being the only one unable to cope". Some respondents considered that the support they received was *empowering *and that it engendered *hope *with one individual explaining that "constant reminders" of them being "close by, loving, and knowing who you were 'before'... really helped me to know one day I would feel happy again to be alive".

Some respondents mentioned the role of seeking support in maintaining and strengthening positive *relationships*. Finally, in some cases *prayer *was mentioned as an advantage of seeking help from family and friends.

#### Informational support

The most common form of informational support was provided in the form of *advice*. One respondent commented: "The main advantage of talking to family and friends is that one can discuss your problems with others and get their opinions about your problems and how to solve your problems". Respondents valued the 'practical' or 'real life' aspects of the advice they received from family and friends. Some respondents particularly valued advice offered by someone with personal experience with depression, citing the importance of being able to *mutually share experiences *with a friend or family member and to receive guidance to find suitable help.

Indeed a commonly reported form of informal informational support involved *facilitating help seeking from a formal source*. Many described the process in terms suggestive of gentle encouragement and guidance. For example, one respondent commented that "a close friend was so helpful and guided me to my GP from there I started to regain my health". However, others used terms suggestive of a less subtle process in which the family member or friend more forcefully directed the help seeking process or even assumed the decision making role. For example, one respondent stated that "Family convince you to seek help!!" and another that: "My wife was able to make the decision for me to go see a GP... I could not have done this as I did not understand what was wrong with me. Had nothing been done I would probably have attempted to harm myself".

Family and friends also provided feedback that assisted the person in evaluating their current situation (*appraisal support*). For example, one respondent commented that the advantage of seeking help from family and friends "is getting their perspective of the whole problem, making comparisons". Respondents often referred to discussions with family or friends as enabling them to see their life from 'a different perspective' and as enabling them to 'refocus', 'to see things more clearly'; or to 'provide some grounding/reality check'. Some respondents indicated that this assisted them to perceive their situation 'more positively'. Conversely, a number of respondents reported that family or friends were able to assist them to recognise that they were depressed and that they needed professional help. For example, one respondent stated: "A close friend or family member would notice my changes in behaviour/thinking and advise me to seek professional help".

#### Companionship support

The next most frequently cited social support sub-category was companionship. The most commonly reported type of companionship support related to satisfying a need for *connection*. As one respondent noted: "I didn't feel alone once I had told them how I was feeling". A small number of people also cited *humour *as an advantage of seeking informal support.

#### Instrumental support and willingness to take positive action

Respondents mentioned *specific practical support *as an advantage of seeking help from family and friends. As noted by one respondent, family and friends "can provide you physical support to survive day to day". Within this subcategory a number of respondents mentioned the importance of others supporting them by caring for their children and/or undertaking their share of household duties. Other respondents referred in general terms to *a willingness *by family and friends to take *positive action or to help *'in practical ways'.

#### Universality

Respondents reported advantages in speaking to a family member or friend who had themselves experienced depression. For example, one respondent referred to helpfulness of "finding out that many people also shared experiences with PND and I was almost in the majority and not the minority!!", and another its helpfulness "to overcome feelings of loneliness, inadequacy, of being the only one unable to cope".

### (ii) Category 2-Background knowledge

Many respondents cited that an advantage of seeking informal support was that family and friends already had important background knowledge about them. Two subcategories emerged. The first was that family and friends knew the respondent individually; the second was that they were aware of the respondent's personal circumstances.

#### Knowledge of the person

The fact that family/friends knew the person well was seen as providing helpful insights into the respondent and what might help them. For example, one respondent commented that friends and family: "know you on a more intimate basis and thus have some common grounding or basis on which they know what makes you tick and something of your beliefs, ideals and tribulation" and another wrote that "them knowing you and your situation intimately gives them further insight into solving your problems". In some cases this knowledge was seen as providing "insights ... that a professional could not know". In particular, knowing the person before they became depressed was cited as important by a number of respondents. As one respondent wrote: "They can carry a sense of the 'you' you have lost for the time being". This placed family members/friends in a position to recognise the problem and to understand its true severity and impact.

#### Awareness of circumstances

A number of respondents believed that it was an advantage that family and friends were familiar with the respondent's circumstances and the potential causes of their depression. It was thought that this knowledge helped the family member to better understand the respondent's problem. This was seen as an advantage because it relieved the respondent from the necessity of explaining the situation: "They knew the circumstances, personal history and background so there was less information to convey to them". It was also seen as placing the family member or friend in a better position to assist the respondent to cope with the precipitating circumstances: "They understood what caused me to be depressed in the first place and helped me to cope with the situation around me at the time".

### (iii) Category 3-Offloading burden

Many participants reported that it was an advantage to offload their burden by talking to family and friends. As one respondent wrote, it is "always helpful to talk about a problem. Things sometimes don't seem so bad when you talk about them". This process was described in various ways such as bringing 'feelings out in the open', 'getting things off my chest', 'offloading excess baggage', 'talking things out', 'giving a voice to it', and 'being able to share the burden'. Simply talking about the problem was often described as providing some relief: "It is important to speak to others because it feels like a heavy weight has been lifted". In other cases talking about the problem led to assistance. As one respondent explained: "Talking out problems helped and family and friends tend to help when they know a problem exists. If they don't know they can't help".

### (iv) Category 4-Personal attributes of the friend or family member

The personal attributes of the source of informal support emerged as a further important perceived advantage of seeking help from friends and family members. *Trustworthiness *was seen as a key attribute of family and friends by a number of participants, one of whom explained: "You know you have their trust and can be quite candid about your problems. They are interested in your well being. You are part of a 'group' with which you are familiar and trusting".

Some participants emphasised that they valued their relatives' or friends' *honesty *and some that their friends and family members were *non-judgmental*. Other participants appreciated the fact that their family and friends were sincerely interested in their *well being and best interests *and that they were *genuinely caring *"enough to be there (without being paid!)". Another valued characteristic was that they were *loving*. Finally, a small number of participants described the family member or friend as a *good listener *or *patient*. One respondent reported: "They don't mind how long it takes e.g. months years-they are always going to give supportive time".

### (v) Category 5-Accessibility and acceptability of help

The accessibility of family and friends was seen as an advantage by many participants. In contrast to professionals, family and friends were perceived as *approachable*, *available whenever they were needed *'day or night', *without time restriction*, and at *no cost*. Other advantages were that it was possible to discuss problems *honestly and openly *with family and friends, being able as one respondent put it to "expose dark feelings to the light". Some respondents also felt *less embarrassed *and *less shame *when seeking help from family or friends. For example one respondent reported that they were better able to accept "the feelings of inadequacy and vulnerability" than if they had "consulted others outside of my immediate family unit". Some participants reported that they were *more comfortable *talking to family and friends, noting that it was possible to *be yourself *and that the help was more warm and *personal*.

### (vi) Category 6-Education of the friend or family member (n = 30)

A substantial minority of participants indicated that an advantage of speaking to family and friends was that it increased the latter's level of *understanding *of the problem, and provided an explanation for the participants' altered behaviour. This was often seen as helpful for the family member themselves. As one respondent noted: "They knew that I was going through a bad time and not to take things personally". However, in some cases this increased understanding was seen as a benefit to the person with depression. For example, some participants identified that it led to *increased tolerance *by the family member, allowing them "to understand what is happening to you, rather than criticise you for the differences you portray due to the depression". Others reported that informing close-others of the problem resulted in more support, because they were then "aware of what you are going through and can give support and share the burden".

### (vii) Category: Other

A number of responses could not be coded into the above categories. Some participants made broad *general *comments about the advantages of seeking help from family and friends: "You feel better", "Very helpful", "Absolutely vital". Others cited specific advantages that fell outside the six categories. Thus, some participants indicated that family and friends provided a *listening ear *with one participant stating: "Just talking to someone who is willing to listen was the key". Several participants emphasised the importance of *openness and self-disclosure*. Finally, some commented on the usefulness of seeking help from a particular family member or friend with *health professional expertise*.

### Disadvantages

As shown in Table 2, eight broad disadvantages were identified from the content analysis of the brief responses. These were in order of decreasing frequency: (i) stigma (n = 53); (ii) inappropriate support (n = 45); (iii) lack of knowledge, training and expertise (n = 32); (iv) adverse impact on family/friends (n = 20); (v) change in relationship with family or friends (n = 18), (vi) unhelpful personal attributes of family and friends (n = 10); (vii) unhelpful outcomes (n = 14); and (viii) too much or too little accessibility (n = 6).

**Table 2:** Frequency (n) of respondents citing disadvantages of seeking support for depression from family and friends

Categories and subcategories	n	Coded Types
**1. Stigma**	**53**	
Stigmatising responses	37	'Get over it' (n = 16), query/minimise illness (5)*, contempt/scorn/vilification (3), ridicule (1), criticism (2), self-pity (2), selfish (1), attention seeking (1), whinger (1), weak (1), to blame (1), undermine (1), pity (2).
Anticipated stigma from family/friend	11	Fear of being: judged (3), losing respect (2), pitied (1), rejected (1); fear of what others were thinking (2)
Self stigma	7	Feeling: burden to others (4), embarrassment (1), shame (1)
2. **Provided inappropriate/inadequate support**	**45**	
Insufficient support	30	Informal sources: lack emotional understanding (14), dismiss illness (9), lack interest/care (6), lack sympathy (3), egocentric responses (2), ignore (1)
Decreased self-esteem	6	Sense of: inadequacy (4), worthlessness (2), uselessness (1), dependency (1)
Over-involvement	5	Too much advice (3), boundary problems (1), worrying excessively (1)
Lack of confidentiality	5	Confidentiality breach (5)
**3. Lacked knowledge, training, expertise**	**32**	Lack of: understanding of depression (12), professional knowledge/training (12), knowledge about helpful actions (5), experiential knowledge (3), objectivity (3)
**4. Adversely affected family/friends**	**20**	Burden on family/friend (7); cause family: stress (4), concern (3), frustration (3), self-esteem problems (3), negative affect (3), upset (2), confusion (1)
**5. Adverse change in relationship**	**18**	Relationship: altered (10), lost (or in danger of being lost) (5), overly supportive (1), unbalanced (1), strained (5).
**6. Negative personal attributes**	**10**	Judgmental attitudes (6), lack of objectivity (6)
**7. Unhelpful/harmful**	**14**	
**8. Accessibility problems**	**6**	Lack of separation (2), could not fully confide (2), geographically inaccessible (1), preoccupied with own burden (1)
**9. Other**	**53**	Increased consumer vulnerability (8), family/friend own agenda (6), pre-existing opinions; (4), fear family/friend will lack patience (5), trusting others undesirable (2), self disclosure stressful (2), negative consequences if fail to respond to help (1)

*Numerals in brackets refer to number of participants citing type of disadvantage.

### (i) Category 1-Stigma

The most commonly cited disadvantage of seeking help from family and friends centred around issues of stigma. These included the stigmatising responses of family and friends, anticipated stigmatising responses from these sources, and internalised (self) stigma.

#### Stigmatising responses

Some participants were told by family and friends from whom they sought help that they should just *get over it*. For example, one respondent was told to: "Pull your socks up, get over it, tell someone who cares" and another to "build a bridge and get over it". Some family members would *not accept the validity of the person's depression*. As one respondent explained: "Some denied it or questioned the validity of my experience & explanations for it. It was very disappointing & upsetting to ask for help & not receive it". Other responses reported by participants were *contempt/scorn/vilification, ridicule *and *criticism*. Some participants reported that family/friends believed that the participant was *just feeling self pity*, or was *selfish*, *attention seeking *or a *whinger*. As one participant reported: "Everyone wants to know what's going on and then when you tell them they treat you as if you are a whinger". Other stated disadvantages of self disclosure to family and friends was that they *think you are weak*, that such disclosure can provide 'excuses for others to lay *blame' *and that friends can '*undermine *one's image or distort it'. Finally, participants specifically mentioned the experience of being pitied.

#### Anticipated stigma

Some participants indicated that a disadvantage of seeking informal support was that they were *concerned about what others would think*, and feared being *judged*, *losing the respect *of family and friends, being *pitied *or *rejected*.

#### Self stigma

Internalised stigma (or self-stigma) was also a barrier to disclosing to family and friends. Participants mentioned their concern and guilt that they would '*feel like a burden*' in speaking to them, that they were *'embarrassed *to admit' their problems and would feel *ashamed *about others close to them knowing of their problems. There was also evidence of more subtle forms of self-stigma. For example one respondent reported as a disadvantage of help seeking from family or friends: "Thinking that they would soon be fed up with my 'winging' [sic]". Although somewhat ambiguous (and coded in this study as '*anticipated stigma*'), it might be inferred that the participants themselves believed that they were whingeing and that continued disclosure and help seeking was unreasonable.

### (ii) Category 2-Inappropriate support

The second most commonly cited disadvantage of speaking to family and friends was that the support it yielded was inappropriate. This was primarily attributed to *insufficient support *but in a minority of cases was associated with *over-involvement*, *decreased self-esteem*, and *lack of confidentiality*.

#### Insufficient support

One basis for insufficient support was a *lack of emotional understanding *which as one respondent explained "can make you feel worse". Another involved people who were actively *dismissive of the illness*. For example, one respondent reported that "My mother told me there was nothing wrong with me and I shouldn't be taking antidepressants because I didn't need them" and another that they had "everything going for them" so "what could possibly be wrong!". Participants also reported a lack of *interest *or *care*, lack of *sympathy *and being *ignored*. Two participants mentioned the unhelpfulness of *egocentric *responses from family or friends, one noting that "Some friends take ones "sharing" as the impetus to "share" their own and relate all one has said to themselves-when one is not at the time ready for this".

#### Decreased self-esteem

A disadvantage of some types of support from family and friends was that it resulted in a lowering of self-esteem. As one respondent noted: "It made me feel worthless no-one fully understands". Another wrote: "My family particularly and some friends felt overwhelmed and inadequate and just wanted me to get better, which increased the pressure and feelings of inadequacy that I already had". In particular, participants found that the disclosure led to a sense of *dependency, inadequacy*, *uselessness*, and *worthlessness*.

#### Over-involvement

Some participants cited too much support as a disadvantage of seeking informal help. One respondent wrote: "They felt they should help rather than just listen. They became part of the "drama". The expected regular 'updates'. They found it difficult to 'back off' ". Specific subcategories were family/friends displaying *boundary problems*, *providing advice rather than just listening *(n = 3), and *worrying excessively *about the participant.

#### Lack of confidentiality

A final negative aspect of the support provided by families is that in some cases they breached confidentiality, with one respondent explaining for example that they may "feel obliged to tell other family members of the situation".

### (iii) Category 3-Lack of knowledge, training, expertise

Many participants reported that a disadvantage of seeking support from family and friends was that they lacked appropriate knowledge or training to assist. This included a *lack of understanding of depression *specified in general terms (e.g., "Some people do not understand what depression is"). Others were more specific in identifying *lack of professional knowledge *or training about depression as a problem: "The person was not trained, or aware of the nature of depression. Therefore his approach (whilst compassionate) fed the depression rather than alleviating it". Lack of *experiential knowledge *was also a reported limitation as one respondent explained: "Everyone has their own opinions and I was inundated with words of wisdom from people who had never experienced depression". Others cited *not knowing how to help *as a disadvantage of seeking help from informal sources. Described by one respondent as they "don't usually know what to do", this problem may be linked to lack of training and lack of experiential knowledge. Finally, *lack of objectivity *was cited as a disadvantage. For example, a respondent commented: "They can ... allow their subjective opinions to interfere with their support! They may have vested interests in the subject matter of the depression which could also interfere with neutral help and support".

### (iv) Category 4-Adverse impact on family/friend

A number of participants expressed concern that seeking help from family and friends impacted negatively on those from whom they sought the support. There was concern that they were *burdening *family members/friends with their problems. It was felt that talking to family/friends about depression caused them *stress and anxiety *and *concern*, and that it hurt or *upset *them (e.g., "You know how painful it is for the listener to hear and see you like this"). Respondents perceived that the help seeking process could negatively impact on their informal helper's *mood*. For example, one respondent wrote: "They can feel bad because they want to help or feel they should have known, or should have been able to stop you feeling bad". Some participants reported that the sense of *self-esteem *of the friend/family member had been negatively affected (e.g., "My family particularly and some friends felt overwhelmed and inadequate") and one respondent reported that many confidants had been *confused*. Finally, some participants reported that family and friends reacted with *frustration *when they were unable to understand or provide a solution to the problem (e.g., "They don't like to talk about what's wrong, and get frustrated when I can't explain what's wrong, or why I burst into tears for no reason"; "Frustration in not being able to help").

### (v) Category 5-Adverse change in relationship

Another disadvantage cited by some participants was that seeking help from family or friends impacted negatively on relationships with them. A number of participants reported being *treated differently *as a result. For example, one respondent wrote that "they treat you very carefully, not normally like they used to", and another described a feeling that "they were always watching me to make sure I wasn't going to kill myself". Related to this, a participant described how disclosure led to over support over time: "They want to run your life when you are well and continue to give advice" and another warned that there was the "potential to *affect the balance/dynamic *of [the] relationship permanently". Several participants cited either a *loss of a relationship *or a fear that this might occur. A small number of participants also indicated that seeking help from family could place a burden and *strain *on *the relationship*.

### (vi) Category 6-Negative personal attributes

There were several reports that some family and friends possessed negative personal attributes that served as a disadvantage when seeking their support and assistance. This included reports of *judgmental *attitudes and a *lack objectivity*. With respect to the latter, one respondent commented: "They are often too emotionally involved to give good advice".

### (vii) Category 7-Unhelpful or harmful

A number of participants reported that seeking help from family or friends either did not help or exacerbated their condition. For example, one respondent described receiving "heaps of advice which was neither useful or productive", another noted that "many people don't understand and can make you feel worse", and a third respondent indicated that "someone close to you may fob it off and say it will go away or you'll feel better and the problem may escalate".

### (viii) Category 8-Accessibility issues

Two participants indicated that *lack of separation *was a problem, one for example, finding it a problem that they could not "shut everything out when I feel need to withdraw into myself". Conversely two found that their friends/family were inaccessible, in one case due to *physical separation *and in the other because the family was 'often *busy with their own burdens'*. Finally, two participants felt that they *could not fully confide *in friends/family, being unable to completely share what they described as their 'darkness' 'shortcomings' or 'true' feelings.

### (ix) Category 9-Other

A number of other disadvantages of speaking to family and friends could not be coded into the above categories. Several respondents indicated that self-disclosure placed the consumer in a position of *vulnerability *(e.g., "Letting walls down to someone close can make you feel very inadequate and vulnerable"). There were also issues of trust (e.g., "I find it undesirable to disclose negative personal information to anyone"). Further, it was *stressful and difficult to disclose *one's mental health status and there was fear that the family or friend may lack the requisite patience. Some participants indicated that friends and family had their *own agenda*. For example, one respondent reported that a "close friend consulted for support was also [a] work colleague who used it to his advantage in the work place" to achieve a career gain. Another respondent cautioned that family and friends "may have vested interests in the subject matter of the depression which could also interfere with neutral help and support". Some family and friends were perceived as *biased by pre-existing opinions*. One respondent for example, described the difficulty caused by friends who "believe I should look to "alternative" remedies & counselling-they are very anti-medication-this is very irresponsible because in my case the only thing that has worked is medication and I would not be here if it were not for my treatment-people can be judgemental". Finally, one respondent indicated that there were "*negative repercussions *from those closest to you *if you don't respond to their help*".

## Discussion

This is the first systematic study of the advantages and disadvantages of informal help seeking for depression. The absence of previous studies is surprising given the importance that consumers with depression and the general public place on informal help seeking [1,2,6] and in view of the low prevalence of timely, formal help seeking among people with depression [16,17].

Overall, people who sought help from family or friends for depression were more likely to report advantages than disadvantages of this help seeking with over 80% citing an advantage. Nevertheless, almost 40% of participants reported at least one disadvantage of seeking informal support indicating that although consumers tend to find help seeking from informal sources helpful, it is not without its risks.

### Advantages of informal help seeking

The primary advantage of consulting family and friends was the support they provided, particularly in the form of emotional and informational support, but also in terms of companionship and instrumental support. These findings are consistent with a broader body of social support literature which has identified them as part of a typology of social support (e.g., see [18]). They are also consistent with the types of social support which have been observed in face-to-face and online peer-to-peer interactions in mutual support groups for depression [19,20].

The most commonly cited type of positive support obtained from family and friends was emotional support, a finding which is consistent with Vollmann et al.'s [11] observation that consumers anticipated emotional support would be the most helpful type of social support for depression. On the other hand, in the current study, informational support, in the form of advice, facilitation of help seeking, appraisal support, and experiential sharing was the second most frequently cited advantage of social support, whereas the consumers in Vollmann et al.'s study anticipated that tangible support would be more helpful than informational support [11]. These apparently divergent findings are not necessarily inconsistent, however. First, whereas Vollmann et al. investigated the relative perceived *value *of different types of support, the current investigation examined the relative *frequency *with which it was reported. Those who provide informal support may fail to perceive the need to provide particular types of support such as instrumental support to a person with apparently adequate physical abilities despite its potential value to the recipient. It is possible then that only a minority of participants in the current study received instrumental support with the consequence that only a minority could report its advantages. Had the current study sought to measure participants' beliefs about the likely helpfulness of different types of support, whether or not they had received such support, the results may have more closely resembled those of Vollmann et al. Alternatively, the severity of depression may have been lower among the current participants recruited from the general community compared to those recruited by Vollmann et al. [11] from a self-help group and psychiatric centre. If tangible support is more helpful for those with more severe symptoms the discrepancy in the two studies could reflect a difference in symptom severity in the two samples. A third possibility is that in contrast to the current community sample, the informational needs of many of the participants recruited in the Vollmann et al. [11] study had been satisfied by formal sources and peers in their mutual support group.

Vollmann et al. [11] reported that consumers anticipated that 'protection oriented support' would be more helpful than 'activation support'. Neither types of support emerged as strong subcategories in the current study. 'Safety' which incorporated the concept of protection oriented support was mentioned by only three participants and examples of activation support were rarely mentioned in the current study.

On the other hand informal confidants can (and do) provide informational support which facilitates formal help seeking. Previous research suggests that the majority of members of the community who experience an episode of depression do not receive professional help at the time of the onset of the illness and that the delay in receiving such help is on average, 8 years following the first episode of depression [16]. Family members and friends are in a position to facilitate earlier help seeking which in turn may improve the longer term outcomes and decrease recurrences of depression.

Nevertheless, currently, there remain barriers to help seeking from formal sources including stigma [13], insufficient mental health professionals [21] and time-poor general practitioners who typically do not provide the psychological treatments preferred by many consumers [22]. Further, depression by itself can render communication difficult. The perceived trustworthiness and non-judgmental attitudes of family and friends, their accessibility and their familiarity with the consumer's circumstances may facilitate communication and make it easier for a person with depression to seek informal than formal help.

A final important perceived advantage of seeking support from family and friends was the possibility that this would increase the latters' awareness and understanding of the problem and ensure that they did not misinterpret the consumers' actions. This has potential advantages for both the carer and the consumer. The changes in social behaviour associated with depression can be marked and may ultimately be associated with the alienation of friends and family and a consequent loss of their social support. This in turn may have further negative consequences for the mental health of the person with depression, potentially resulting in slowed recovery or exacerbation of symptoms.

### Disadvantages of informal help seeking

Not all consumers reported that seeking help from friends or family members resulted in helpful emotional and informational support. Nor were families and friends universally perceived to be non-judgmental about depression.

The most frequently cited disadvantage of seeking informal support related to actual, perceived and internalised stigmatising responses. These were encountered by a substantial minority of consumers who sought help for depression from informal sources. The moderately high prevalence of stigmatising attitudes toward depression in the community has been documented previously [23,24] and perceived levels of depression stigma are very high [23,24]. It is not surprising then that some family members hold negative attitudes to depression and that in the current study stigmatising responses and anticipated stigma were seen as a disadvantage of help seeking from family. Thus, although some consumers in the current study reported that family members were non-judgmental, and although stigma has previously been found to be lower among family members and those with the most contact with people with depression [24], it is clear that stigma is a problem in some families. Such stigma may be particularly toxic for several reasons. First, interactions with family members and friends are likely to be the more frequent than other interactions and thus exposure to stigma from these sources may be particularly critical. Secondly, stigma can be conceptualised as a stressor and perceived stigma is known to predict depressive symptoms [25] among those with a mental disorder. Social support may mitigate the effects of stress in producing depression, but most social support is sourced from family and friends. Thus, whereas family and friends may serve to protect consumers from the negative outcomes of exposure to stigma originating in members of the community, no such protection is available if the stress arises from stigma originating from family or friends.

Although many consumers perceived informal social support as an advantage, some participants considered that friends and family provided too much support or support which was unhelpful. Such support may serve as a stressor [26] and there is evidence that it can exacerbate 'negative feelings', at least in those with physical conditions [27,28]. Some consumers reported a lowering in self-esteem due to a sense of dependency and inadequacy as a result of receiving social support. However, other consumers in the current study reported a negative effect, including lowered self-esteem, due to insufficient or no support arising from a lack of understanding or a dismissive attitude (see stigma above).

Although informational support was valued by many consumers in this study, others perceived lack of knowledge and expertise as a disadvantage of this type of support. Such lack of understanding has previously been reported as a limitation of help seeking from informal sources [21]. Moreover, Vollmann et al. [11], documented discrepancies between the illness representations of people with and without depression, noting that such differences underpin "problematic communications and conflicts in social interactions between depressed and never depressed persons" (p. 5).

### Implications

Mental health educational programs for the public are common and there is evidence that they can improve knowledge of mental illness and decrease the stigma associated with these conditions [29-31]. However, such programs rarely target caregivers specifically. The current study findings suggest that consideration should be given to developing and evaluating the effectiveness of public educational and destigmatisation programs designed for the caregivers of people with depression. Such programs could be delivered by means of a variety of dissemination strategies such as face-to-face lectures, brochures, and the Internet. They should aim to increase carer depression literacy, incorporate stigma reduction messages and assist carers to understand how they might facilitate help seeking and provide emotional support in a respectful way that is sensitive to personal boundaries and issues of self-esteem. A key component of such an educational program would be information about what consumers find helpful and what they find unhelpful. The current findings would provide a basis for this part of the program. This might include conveying the value which consumers place on social support including emotional support (understanding, love, caring, the importance of privacy and confidentiality); well informed informational support (eg, assistance with information about sources of help); companionship and instrumental support. At the same time, based on the current findings, the program would discuss the types of support that *can *be counterproductive such as overinvolvement. Such program should also assist family and friends to understand that the negative behaviours are due to the illness rather than a rejection of them. It would further involve an explanation of the different types of stigma (including internalised stigma) and their consequences and the negative impact they may have on recovery, and the delivery of an evidence-based destigmatisation program. Other components would include education about the nature of depression and its symptoms and consequences, and information about which treatments work and the consequences of neglecting treatment. An equally important component of such a course would be the incorporation of strategies for supporting the mental health of the carer.

### Limitations

The study investigated the advantages and disadvantages of seeking support for those participants with depression who sought help. However, the study did not seek input from the people with depression who opted not to seek help from family and friends. This group may have avoided seeking help due to perceptions of the disadvantages of doing so. Conceivably some of these perceptions were well founded (e.g., friends and family held known stigmatising attitudes to depression or were socially unsupportive). Thus, the current findings may have underestimated the extent of the disadvantages of help seeking from informal sources. In addition, the study relied on an open ended question and the coding was undertaken using a consensus approach. The quantitative data in this paper is therefore only broadly indicative of the issues experienced by respondents. A final limitation of the current study is that like that of Vollman et al, it did not investigate whether the perceived advantages and disadvantages differed for consumers with different demographic or clinical characteristics. It is possible that the perspectives of men and women, and people of differing ages, educational, social and cultural backgrounds, severity and length of depression demonstrate differing patterns of response. The nature of the relationship of an informal carer to the consumer and caregiver characteristics may also be important factors in determining the perceived benefits and costs of informal help seeking. The categories generated in the current qualitative study can serve as a basis for developing items for a structured survey that investigates quantitatively the independent role of these factors and their interactions in predicting consumer-perceived advantages and disadvantages of informal help seeking. Finally, future research should investigate the information needs of carers and consumers from different backgrounds and the relative efficacy and acceptability of broad compared to targeted educational interventions for these groups.

## Conclusions

Family and friends are well placed to provide support and to facilitate help seeking for depression. However, some family members/friends may be unhelpful or even toxic and there may be benefits in undertaking community educational and destigmatisation programs which target carers. Further research, including quantitative surveys, is required to investigate the role which family and friends play in supporting people with depression in the community as are pragmatic trials of the effectiveness of suitably designed educational interventions for carers.

## Competing interests

The authors declare that they have no competing interests.

## Authors' contributions

KG conceived the study, developed the open-ended item, developed the data coding system, and wrote the first draft of the paper. DC developed the data coding system, undertook the final coding of the data and edited the paper. LB designed and implemented the survey, and critically reviewed and provided editorial comments on the paper. RR undertook the final coding of the data and provided editorial comments on the paper. All authors read and approved the final version.

## Pre-publication history

The pre-publication history for this paper can be accessed here:

http://www.biomedcentral.com/1471-244X/11/196/prepub
